# An Energy-Balanced Clustering Protocol Based on an Improved CFSFDP Algorithm for Wireless Sensor Networks

**DOI:** 10.3390/s18030881

**Published:** 2018-03-16

**Authors:** Yiming Zhang, Mandan Liu, Qingwei Liu

**Affiliations:** School of Information Science and Engineering, East China University of Science and Technology, Shanghai 200237, China; yiming227@163.com (Y.Z.); liuqingwei@mail.ecust.edu.cn (Q.L.)

**Keywords:** wireless sensor networks (WSNs), clustering, CFSFDP, energy-balanced protocol

## Abstract

Clustering, as an essential part in an hierarchy protocol that can prolong the network lifetime, is influenced by the cluster head selection and clustering scheme. A new clustering algorithm called clustering by fast search and finding of density peaks (CFSFDP) based on local density and distance is implementable and efficient. In this paper, we combine this clustering algorithm with a hierarchy protocol in wireless sensor networks (WSNs). However, energy consumption in each round is unbalanced only considering these two variables during the clustering phase, which leads to the early death of the first node. In order to solve this problem, we take residual energy into consideration in our improved CFSFDP-E (energy) algorithm so as to ultimately balance the energy consumption of the network. We analyze different forms of energy and choose a dynamic threshold for each round in the CFSFDP-E algorithm. Simulation results demonstrate that the proposed approach can not only postpone the death of the first node by almost 50% compared to LEACH, but that it also outperforms several related protocols with respect to energy efficiency.

## 1. Introduction

Wireless Sensor Networks (WSNs) are composed of a large number of multifunctional low cost sensor nodes with restricted battery power supplies, which are randomly deployed inside the sensing field [1]. Since WSNs are usually deployed in a hostile environment without supervision, it is complicated to recharge or replace the batteries when out of energy. Therefore, energy efficiency and network lifetime become an emphasis in WSNs. Due to the fact most of the energy of sensor nodes is consumed by data transmission, an energy-efficient routing scheme should be utilized to effectively reduce the energy consumption. Clustering, as an essential part in any hierarchy protocol, can prolong the network lifetime, and is influenced by the number of clusters, cluster head selection and clustering scheme. Sensor nodes are grouped by clustering algorithms. Each cluster contains one cluster head (CH) and some normal cluster member (CM) nodes. CMs transmit the data to their own CH which has the responsibilities of receiving, aggregating and forwarding these data to a base station (BS). When a node is out of energy, it is defined to be dead. The round when all the nodes have died is the network lifetime. As the sensed data has to be forwarded to the BS for further necessary action, routing becomes important for transferring data from node to node or to the BS efficiently [2]. Therefore, it is indispensable to balance the energy consumption among sensor nodes to prolong the network lifetime for clustered WSNs.

There have been lots of clustering algorithms and schemes applied to WSNs. KM-LEACH [3] uses the K-means clustering algorithm to form clusters. Reference [4] implemented both centralized and distributed K-means clustering algorithms. LEACH-CKM [5] takes the remote nodes into account during the formation of groups by the K-means classification method and uses a Minimum Transmission Energy routing protocol to transmit information. In addition to the application of K-means, many factors are considered in other algorithms. Reference [6] proposed a new method called CAST-WSN based on the node distance from the gravity center of the cluster, the node distance from the energy center in each cluster and a Steiner tree structure, which brings significant improvements to the C-Means clustering algorithm. Besides residual energy, the distance from the nodes to the base station, neighbors and the number of neighbors are considered for cluster head selection [7]. Distributed unequal clustering using fuzzy logic (DUCF) takes residual energy, node degree and distance to the BS into account to ensure load balancing among the clusters by varying the cluster size [8]. Moreover, some other theories and algorithms are adopted in WSNs, such as game theory. Each sensor node is considered as a rational and selfish player which will play a clustering game with an equilibrium strategy. This approach is proved to have a good energy balancing performance and consequently the network lifetime is greatly enhanced [9].

In this paper, we implement a new clustering algorithm in WSN which is called clustering by fast search and finding of density peaks (CFSFDP) [10] published in *Science* in 2014. It is an implementable and efficient algorithm with two variables: local density and distance. It divides clusters based on the idea that cluster centers are characterized by a higher density than their neighbors and a relatively large distance from points with higher densities.

This algorithm has been applied in some other areas, for example, reference [11] applied terahertz time-domain spectroscopy (THz-TDS) combined with CFSFDP for pesticide detection. To solve the similar distance problem in THz spectra data, they proposed PCA-CFSFDP. Principal component analysis (PCA) was used to reduce the dimensions of THz spectrum data, and then the PCA result was used as the input of CFSFDP. The experimental results showed that PCA-CFSFDP approach can get a satisfactory detection result. Reference [12] proposed a novel research scheme using density-based clustering and a backtracking strategy. In order to isolate the targets from noises, CFSFDP is employed to cluster targets by the spatial intensive distribution. Compared with several state-of-arts methods, this algorithm is more effective for dim targets with lower signal-to clutter ratio (SCR). Since this clustering algorithm has not been applied in WSNs so far, we adopt in this work in a WSN to choose cluster heads and form clusters. Our main contributions can be listed as follows:

- We apply the CFSFDP algorithm to a WSN to choose cluster heads and form clusters.
- We take nodes’ residual energy into consideration in CFSFDP-E algorithm to balance the energy consumption. The original CFSFDP uses the information of local density and distance to group data points in the dataset. However, all the nodes have limited energy in a WSN. Considering this unique attribute, we should take residual energy as a third key factor in the clustering process.
- Different forms of energy to get a better performance are discussed and we use the value of local density, distance and residual energy after normalization as three main variables in the clustering scheme.
- Since the clustering process needs to be repeated in every round, we set a dynamic threshold for cluster head selection and it can determine an appropriate number of CHs and make every CM be clustered reasonably.
- With the same simulation environment and parameters, it is proved that our proposed CFSFDP-E algorithm in WSN outperforms the classical protocols (LEACH [13], LEACH-C [14], PEGASIS [15] and SEP [16]), some improved protocols based on LEACH (ALEACH [17], C-LEACH [18] and K-LEACH [19]) and some new protocols which were proposed in the last three years (KM-LEACH [3], DBCH [20] and EESCA [21]).

The rest of this paper is organized as follows: Section 2 summarizes some related clustering protocols in WSN. The theory of the CFSFDP algorithm is described in Section 3. Based on CFSFDP, Section 4 proposes the implementation of our improved CFSFDP-E algorithm in WSNs. In Section 5, we compare the performance of CFSFDP-E with those of several other protocols. Finally, we make a conclusions and suggest potential future work in Section 6.

## 2. Related Works

The most classical hierarchical routing protocol in WSN is Low Energy Adaptive Clustering Hierarchy (LEACH), proposed by Heinzelman [13]. In this protocol, cluster heads are randomly selected using a threshold and other nodes choose the nearest cluster heads to form clusters. LEACH incorporates data fusion into the routing protocol to reduce the amount of information that must be transmitted to the base station. However, since the rotation method may cause the hot spot problem, a number of new protocols based on LEACH has been proposed to balance energy consumption and prolong the lifetime of WSNs.

Threshold sensitive Energy Efficient sensor Network (TEEN) [22] is a routing protocol for enhanced efficiency in wireless sensor networks. It sets two thresholds: a hard and a soft threshold. The hard threshold tries to reduce the number of transmissions by allowing the nodes to transmit only when the sensed attribute is in the range of interest. The soft threshold further reduces the number of transmissions by eliminating all the transmissions which might occur when there is little or no change in the sensed attribute according to the hard threshold. Power-efficient gathering in sensor information systems (PEGASIS) [15] is a chain-based protocol that is an improvement over LEACH. In PEGASIS, each node communicates only with a close neighbor and takes turns transmitting to the base station, thus reducing the amount of energy spent per round.

To avoid uneven distribution problem of cluster heads and cluster sizes in LEACH, reference [14] proposed the LEACH-Centralized (LEACH-C) algorithm. LEACH-C uses a centralized clustering algorithm and the same steady-state phase as LEACH. During the set-up phase of LEACH-C, each node sends information about its current location and residual energy level to the sink node. Thus, it can choose cluster heads with high residual energy. However, both of them are centralized approaches and not suitable for large-scale networks. To avoid cluster heads’ premature death in LEACH, reference [23] proposes the V-LEACH algorithm. V-LEACH is a new version of LEACH protocol to reduce energy consumption. The main concept of V-LEACH is that, besides having a cluster head in the cluster, there is a vice-cluster head that takes the role of the cluster head when the cluster head dies, so cluster nodes can send data to the base station without the need to select a new cluster head each time, which can prolong network lifetime.

On the other hand, some heterogeneity-aware protocols such as SEP [16] and DEEC [24] are specially designed for heterogeneous WSNs. SEP is aimed at prolonging the stability period of two-level heterogeneous networks, which consist of two types of nodes according to the initial energy, i.e., normal nodes and advanced nodes. SEP works in the same way as LEACH, but for SEP, the CH rotating epoch and election probability are directly related to the initial energy of nodes. As opposed to SEP, DEEC further improves the functions of election probability by considering both the initial and residual energy of the network. It achieves better performance than SEP and LEACH in a multi-level heterogeneous WSN. Unfortunately, DEEC can’t be used when the sink node is located far from the sensor nodes since it is working under the assumption that sink node is located in the center of the WSN.

In recent years, there have been lots of improvements on the original LEACH protocol. ALEACH [17], C-LEACH [18] and K-LEACH [19] improve the cluster head selection, which take nodes’ residual energy, initial energy or optimal cluster numbers into consideration. Raja and Samundiswary proposed the Self-Organized Tree-Based Energy-Balance (STEB) routing protocol [25] in which a sink node assigns a root node and broadcasts this selection to all sensor nodes. Each node selects its parent by considering only itself and its neighbors’ information. STEB outperforms LEACH in terms of rounds and remaining energy. A fuzzy-logical-based clustering approach called LEACH-ERE [26] uses an extension to the energy prediction to prolong the lifetime of WSN by evenly distributing the workload. In addition to the residual energy, the expected residual energy has been introduced to act as a fuzzy descriptor during the cluster head selection process.

There are also some new algorithms and new protocols applied in WSNs. The harmony search algorithm (HSA) [27] is music-based meta-heuristic optimization method which is analogous with the music improvisation process where musician continue to polish the pitches in order to obtain a better harmony. Raval [28] applied it for minimizing the intra-cluster distance and optimizing the energy consumption of the network. IHSCR [29] was an energy-efficient clustering and routing based on the improved harmony search algorithm. It combines a discrete encoding scheme of HAS and the roulette wheel selection method. A three-level hybrid clustering routing protocol algorithm (MLHP) [30] based on the grey wolf optimizer [31] is proposed which use the bionics algorithm for probabilistic selection of CHs. In addition, PSO-ECHS [32] is an energy efficient cluster head selection algorithm based on particle swarm optimization for wireless sensor networks. It considers intra-cluster distance, sink distance and residual energy of sensor nodes, which demonstrated the superiority for prolonging the lifetime of WSN. In order to solve energy bottleneck problem, Wang [33] proposed a pair-wise directional geographical routing (PWDGR) strategy. The source node sends the data to the pair-wise node around the sink node, which can effectively relieve the serious energy burden around sink node and also make the energy consumption balanced. Besides, reference [34] proposed an energy-efficient multi-hop routing algorithm based on grid clustering to tackle this problem. This algorithm optimizes the electoral process of functional nodes by combining various factors such as nodes’ energy, nodes’ location and levels of the network area. Communication nodes are introduced to select cluster heads and transfer data between clusters via multi-hop routing, easing the burden of cluster heads. To balance the energy among clusters, River Formation Dynamic based multi-hop routing protocol [35], which was integrated with clustering and a new hybrid technique, improves energy conservation by reducing the overall packet transmission distance of intra- and inter-cluster communication, which results in increased overall network lifetime.

## 3. CFSFDP Algorithm

The CFSFDP algorithm, proposed by Rodriguez and Laio, forms the basis of a clustering procedure in which the number of clusters arises intuitively, and outliers are automatically spotted and excluded from the analysis. Clusters are recognized regardless of their shape and the dimensionality of the space in which they are embedded. The nodes with higher local density and larger distance will be chosen as cluster heads.

For each data point $x_{i}$, two variables: local density $\rho_{i}$ and distance $\delta_{i}$ will be obtained through CFSFDP. The local density can be calculated by two ways [10]. One is by cut-off kernel:

(1) $$\rho_{i}=\sum_{j\in I\backslash \{i\}}\chi (d_{ij}-d_{c})$$

(2) $$\chi (a)=\left\{ \begin{array}{ll} 1 & a<0\\ 0 & a\geq 0 \end{array}\right.$$

where I=(1,2,…,N) represents the set of indices of data points. N is the number of data points. $d_{ij}$ is the Euclidean distance between point i and point j. $d_{c}$ represents cutoff distance. Equations (1) and (2) indicate that $\rho_{i}$ is equal to the number of points of which the distances to data point i are smaller than $d_{c}$. The other way to calculate local density is by Gaussian kernel:

(3) $$\rho_{i}=\sum_{j\in I\backslash \{i\}}e^{-{\left(\frac{d_{ij}}{d_{c}}\right)}^{2}}$$

The results of these two ways suggest that the larger the number of points in its neighborhood is, the higher the value of local density will be. The difference between them is that the value of cut-off kernel is discrete while that of Gaussian kernel is continuous. 

The other important variable is distance which is based on the local density. Sort all the $\rho_{i}(i=1,2,\ldots ,N)$ in descending order, then the distance $\delta_{i}$ of the point with highest local density $\rho_{\max}$ is set to the maximum value of distance $d_{\max}$. The distance of other points are set to the minimum distance between the point i and any other points with higher density. The expression of distance is defined as Equation (4) [10]:

(4) $$\delta_{i}=\left\{ \begin{array}{ll} \max(d_{ij}) & if\rho_{i}=\rho_{\max}\\ \underset{j:\rho_{j}>\rho_{i}}{\min}(d_{ij}) & if\rho_{i}\neq \rho_{\max} \end{array}\right.$$

As mentioned above, the data points with high distance δ and relatively high local density ρ are regarded as the cluster centers. In order to determine the number of cluster centers accurately, Alex set γ to represent the product of local density and distance [10]:

(5) $$\begin{array}{cc} \gamma_{i}=\rho_{i}\delta_{i} & i\in I \end{array}$$

The data point with $\gamma_{i}$ which is higher than the threshold η will be selected as cluster center. After the cluster centers have been found, each remaining point is allocated to the same cluster as its nearest neighbor with higher density.

CFSFDP, which needs less parameters and no iteration, is highly efficient and effective for clustering non-spherical and unbalanced data and the clustering process is performed in a single step. Due to these advantages, we applied it to a WSN to get a better clustering division and cluster heads selection.

## 4. An Energy-Balanced Protocol Based on the Improved CFSFDP

### 4.1. Energy Model

In this paper, the first order model [13] is used as the energy model of the network. The dissipated energy in the transmitter node ($E_{TX}$) and in the receiver node ($E_{RX}$) with distance d for transmitting an l−bit data packet can be calculated as follows:

(6) $$E_{TX}(l,d)=\left\{ \begin{array}{l} l\times E_{elec}+l\times \varepsilon_{fs}\times d^{2},d\leq d_{0}\\ l\times E_{elec}+l\times \varepsilon_{mp}\times d^{4},d>d_{0} \end{array}\right.$$

(7) $$E_{RX}=l\times E_{elec}$$

where $E_{elec}$ is the dissipated energy (per bit) in either transmitter or receiver circuit, and depends on such electronic factors as digital coding, modulation, filtering and spreading of the signal. l is the number of bits. The distance threshold is defined as $d_{0}=\sqrt{\varepsilon_{fs}/\varepsilon_{mp}}$. The transmission distance d, the free space $\varepsilon_{fs}$ or multipath fading channel $\varepsilon_{mp}$ are used for the transmitter amplifier.

In this model, the use of free space channel model or multipath fading channel model depends on the distance between the transmitter and receiver. If the distance is less than the threshold, the former one will be used, otherwise, the latter one.

Assume that there is one cluster made up with a cluster head node and k cluster member nodes. The transmission distance $d_{toBS}$ between the cluster head and the sink node is larger than $d_{0}$ and the distances between each normal node and the cluster head $d_{toCH}$ are less than $d_{0}$, so, the energy consumption of this cluster head in one round can be calculated as:

(8) $$E_{CH}=l(E_{TX}+E_{DA})+l\varepsilon_{mp}d_{toBS}^{4}+kl(E_{RX}+E_{DA})$$

where $E_{DA}$ represents the dissipated energy (per bit) in data aggregation. $kl(E_{RX}+E_{DA})$ is the energy consumption of receiving packets from k cluster members and data aggregation. $l(E_{TX}+E_{DA})+l\varepsilon_{mp}d_{toBS}^{4}$ is the energy consumption of the cluster head for sending aggregated data to the sink node. The energy consumption of a non-cluster head node is:

(9) $$E_{non-CH}=lE_{TX}+l\varepsilon_{fs}d_{toCH}^{2}$$

where $lE_{TX}+l\varepsilon_{fs}d_{toCH}^{2}$ is the energy consumption of a cluster member node for sending data to the cluster head.

Based on analysis above, the residual energy for node i in the current round r can be calculated by Equation (10):

(10) $$E_{i}(r)=\left\{ \begin{array}{l} E_{i}(r-1)-[l(E_{TX}+E_{DA})+l\varepsilon_{mp}d_{toBS}^{4}+kl(E_{RX}+E_{DA})]\;if\;i\in G_{CH}\\ E_{i}(r-1)-(lE_{TX}+l\varepsilon_{fs}d_{toCH}^{2})\;if\;i\notin G_{CH} \end{array}\right.$$

where $E_{i}(r-1)$ is the residual energy for node i in the r−1 round. $G_{CH}$ represents the set of cluster heads.

### 4.2. Proposed CFSFDP-E Algorithm

In WSNs, the routing protocols can be divided into flat protocols and hierarchy protocols. All the nodes will be divided into several clusters in a hierarchy protocol. For example, in LEACH, cluster heads are selected randomly and the remaining nodes will choose the clusters in which the distance between the node and cluster head is the smallest.

The CFSFDP algorithm can divide data points into clusters according to the local density and distance. Based on this algorithm, we propose an energy-balanced protocol called CFSFDP-Energy (CFSFDP-E) to form clusters in a WSN. In this paper, the premise is that the nodes in the WSN are deployed randomly and each node is location-fixed, energy-constrained and has the same capabilities. The BS is not subject to energy restrictions and has strong communication and computation capabilities. Sensor nodes can be regarded as special data points which have energy attributes. Therefore, it is possible to apply the CFSFDP clustering scheme in the WSN. However, due to the fixed location, the cluster centers will not be changed until the energy of nodes is exhausted. The first dead node will emerge earlier than that in LEACH, so we should take energy into consideration to select the nodes with high local density, high distance and high residual energy as cluster heads.

In order to consider the impact of residual energy, Equation (5) should be changed as follows:

(11) $$\begin{array}{cc} \gamma_{i}(r)=\rho_{i}(r)E_{i}(r)\delta_{i}(r) & i\in I \end{array}$$

where $\rho_{i}(r)$, $\delta_{i}(r)$ is the local density and distance of node i in the current round r of WSN respectively, and $E_{i}(r)$ is the residual energy of node i. The number of cluster heads depends on the threshold η. In the CFSFDP clustering algorithm, the threshold is fixed. However, the threshold must be dynamic to follow the trend of energy changing in improved algorithm when considering energy. We will discuss the dynamic threshold in Section 5.4.

**Figure 1:**
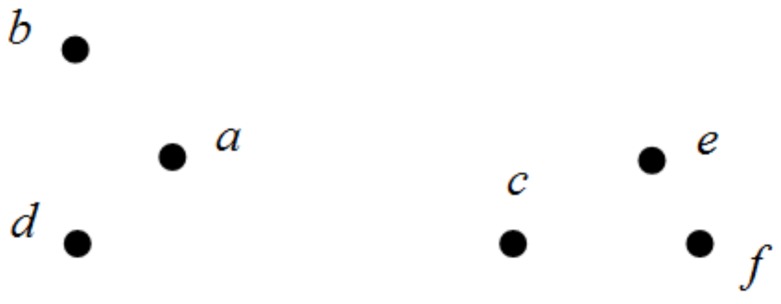
The deployment of six nodes.

The following is an example to explain our proposed CFSFDP-E algorithm. Suppose that there are six nodes a,b,c,d,e,f to be clustered, shown in Figure 1. The local densities are in descending order and the distance of each node shown in Table 1 are calculated by Equation (4). Then, according to the algorithm, since the local density of node a is the largest, the distance of this node should be the maximum value of all the distances. The farthest node from node a is node f. Then according to the descending order of local density, $\delta_{b}$ should be the distance between node b and a node with higher local density, that is, $\delta_{b}$ is $d_{ba}$. Notice that in Table 1, DESC means the local densities are in descending order and the distance of each node is determined by the value of local density.

Suppose that we only choose two nodes as cluster centers. In the CFSFDP algorithm, assuming that two points with the largest γ value are node a and node c, that is, the class of node a is 1 and the class of node c is 2, then other nodes can be clustered based on the descending order of local density. For example, node b choose the same class as the node which has a higher local density and closest to itself. So, the class of node b is 1 which is the same as node a and the class of node d, e, f is 1, 2, 2 respectively.

When considering residual energy in CFSFDP-E, the γ value will be changed. If the two nodes with the largest γ are node c and node b in the CFSFDP-E algorithm, the class of node a obviously cannot be determined because node a has the largest local density and we can’t find a node with higher local density than node a. It doesn’t belong to any class if we still allocate nodes according to local density.

Due to the above problems, we consider how to allocate other normal nodes based on both local density and energy. We define a variable ω called density-energy which can be calculated as follows:

(12) $$\begin{array}{cc} \omega_{i}(r)=\rho_{i}(r)E_{i}(r) & i\in I \end{array}$$

Sort ω value in descending order in Table 2. However, the distance of each node is the same with Table 1, which is still determined by its local density according to Equation (4). Suppose that the nodes with largest γ value are node c and node b, then according to the descending order of ω, we need to discuss about the class of node f. The class of node f should be the same as node e. But the node e hasn’t been allocated to any cluster, resulting in the class of node f can’t be determined, either.

From the analysis above, we can know that CFSFDP-E needs to multiply residual energy into the expression of γ as Equation (11). The allocation of non-CHs should base on the order of density-energy instead of the local density in CFSFDP algorithm. Besides, the value of distance should also be determined by density-energy ω in order to allocate all the nodes correctly. Therefore, the expression of distance should be revised to:

(13) $$\delta_{i}=\left\{ \begin{array}{ll} \max(d_{ij}) & if\;\omega_{i}=\omega_{\max}\\ \underset{j:\omega_{j}>\omega_{i}}{\min}(d_{ij}) & if\;\omega_{i}\neq \omega_{\max} \end{array}\right.$$

Table 3 shows the value of distance according to the ω. The maximum ω is $\omega_{c}$. The farthest distance from node c is node b, that is, $\delta_{c}$ is $d_{cb}$. Other δ values can be calculated through Equation (13). The cluster heads are still node c and node b, so other unclassed nodes can be allocated into clusters according to the descending order of density-energy ω. For example, the distance of node f is $d_{fc}$, which means the nearest node to node f in other nodes that have a higher density-energy is node c. Hence the class of node f is 1, the same as that of node c. Similarly, the class of node d,e,a is 2, 1, 2.

### 4.3. Algorithm Description

The steps of the CFSFDP-E algorithm for each round can be divided into two phases. The first is clustering set-up phase, when all the sensor nodes should be clustered according to the CFSFDP-E algorithm. It will determine the CHs and CMs. The second phase is data transmission. Cluster members send data to their cluster heads and the cluster heads send data to the sink node. From the discussion above, the detailed steps of CFSFDP-E algorithm for one round can be described as follows:

#### 4.3.1. Phase 1: Clustering Set-up Phase

*Step 1*. Parameter Initialization

(1) Set the number of nodes N and maximum round $r_{\max}$ for WSN;
(2) Set cutoff distance $d_{c}$ and threshold η;
(3) Calculate the distance $d_{ij}$ between nodes;

*Step 2*. Determine whether the node is exhausted

Calculate every node’s residual energy through Equation (10). If the energy is exhausted, the node is dead. Only living nodes can go on following steps.

*Step 3*. Select CHs

Calculate every node’s local density, density-energy and distance through Equations (3), (12) and (13) in the current round r, respectively. Based on these parameters, calculate γ of all the living nodes through Equation (11). If $\gamma_{i}>\eta$, the node i will be chosen as cluster head.

*Step 4*. Allocate CMs.

Sort all ω calculated by Equation (12) in decreasing order. Assign the remaining nodes to the same cluster as its nearest neighbor with higher density-energy.

#### 4.3.2. Phase 2: Steady-state Phase

This phase is the same as in LEACH [9]. The cluster head node sets up a TDMA schedule and transmits the schedule to the nodes in its cluster. After all nodes in cluster have received the TDMA schedule, the steady-state operation will begin. All the normal nodes send their data to their own cluster heads and the energy of normal nodes will be consumed in this process. Once the cluster head receives all the data, it performs data aggregation to enhance the common signal and reduce the energy consumption. The aggregated data are sent to the sink node by routing path. Hence the energy consumption of cluster head contains receiving data from cluster members and sending aggregated data to sink node.

After the steady-state phase, the round ends. If total energy has not been exhausted or the current round r hasn’t reached the maximum number of rounds, the next round will choose cluster heads and divide clusters by CFSFDP-E algorithm again, which will repeat Phase 1 and Phase 2.

**Figure 2:**
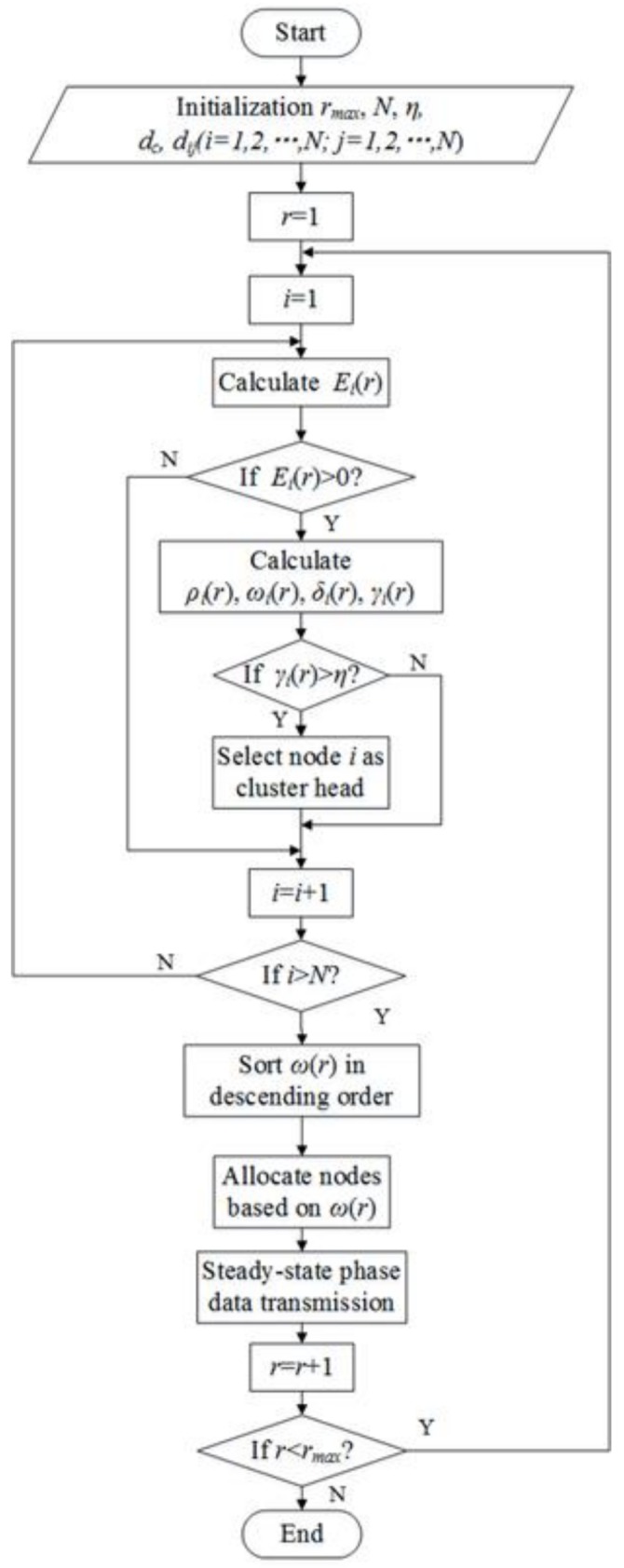
Flow chart of CFSFDP-E.

Figure 2 is the flow chart of CFSFDP-E algorithm implemented in WSN. r represents the current round of the network.

The pseudo-code of the CFSFDP-E algorithm is as follows:

**CFSFDP-E Algorithm**1  **Input**: $r_{\max}$, N, η2  **For** each round r3    **For** each node i4      calculate its residual energy
$E_{i}$5      **If**
$E_{i}$ > 06        the node is still alive, calculate its local density
$\rho_{i}$, distance $\delta_{i}$ and density-energy $\omega_{i}$7        calculate
$\gamma_{i}$8        **If**
$\gamma_{i}$ > η9          select node
i as cluster head10      **end**11     **else**12      the node
i is dead13     **end**14     Sort all the
ω in descending order15     Allocate normal nodes into several clusters based on the value of
ω16     Data transmission phase17   **end**18 **end**

## 5. Experimental Results and Analysis

### 5.1. Operation Environment

The simulation environment is MATLAB R2015a and executed on a 3.40 GHz Intel Core i7-2600U CPU equipped with 4 GB RAM and Windows 10. The main parameters are shown in Table 4. In this paper, the final result of each algorithm is the average result of 20 independent experiments. The number of nodes N is 100.

In the LEACH protocol, the threshold T(i) for cluster head selection is defined as:

(14) $$T(i)=\left\{ \begin{array}{ll} \frac{p}{1-p[r\mathrm{mod}(1/p)]} & i\in G\\ 0 & other \end{array}\right.$$

where p is the probability to be selected as cluster head in each round. G is the nodes set including those nodes which have not yet been selected as cluster head in recent 1/p rounds. rmod(1/p) means the remainder for division of r and 1/p.

### 5.2. Simulation Results of CFSFDP-E Compared with LEACH and CFSFDP

To test the performance of CFSFDP-E, we compare it with LEACH and CFSFDP. Results are shown in Table 5. FND means the first node dies, and HND, LND represent the half of nodes die and last node dies. The network lifetime is defined as the round that all nodes’ energy is exhausted. If the protocol can balance energy well and last dead time is late, it can be defined that the network lifetime is long. In this paper, we assume that the death of 90% nodes means all nodes dead.

We also compare the CFSFDP algorithm in the WSN. It divides nodes into clusters only based on local density ρ and distance δ and the γ value for each node is calculated through Equation (5), while in the proposed CFSFDP-E algorithm, the formation of clusters considers not only these two factors, but also the residual energy of each living node. These algorithms are simulated in the same experimental scenario. The threshold η should be a dynamic value rather than a fixed number to determine the number of CHs. Before discussing about threshold, we choose 10% of living nodes as the number of CHs in each round for both CFSFDP and CFSFDP-E.

From Table 5, we can find that the FND of CFSFDP algorithm in WSN occurs much earlier than that of LEACH. The reason for this result is that the CFSFDP algorithm selects cluster heads with local density and distance, so the location of all nodes is fixed if there is no node to die and the value of local density and distance will not change, which means when a node is selected as CH, it will remain the CH in the following rounds until the energy is exhausted. Thus, the round of FND is 91, which is much earlier than 688 in LEACH. However, the LND in CFSFDP happens later compared with LEACH and CFSFDP-E. It is obvious that the energy consumption of the CFSFDP algorithm is unbalanced.

**Figure 3:**
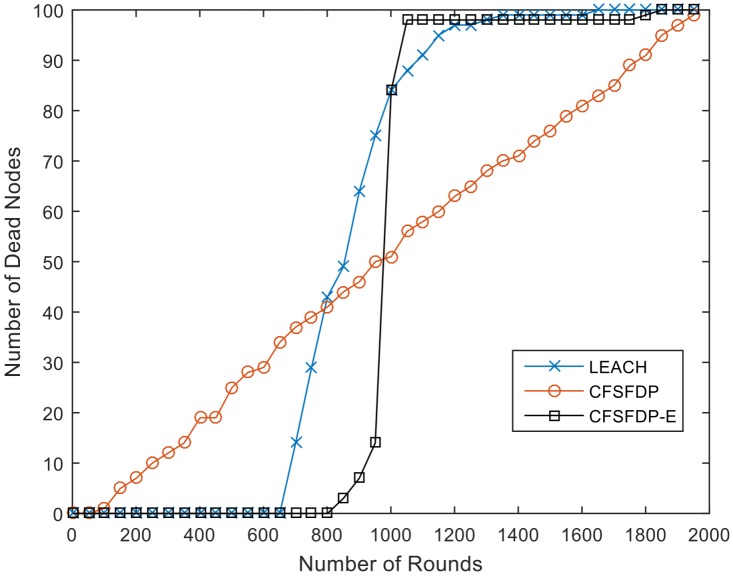
Network lifetime comparison of LEACH, CFSFDP and CFSFDP-E.

**Figure 4:**
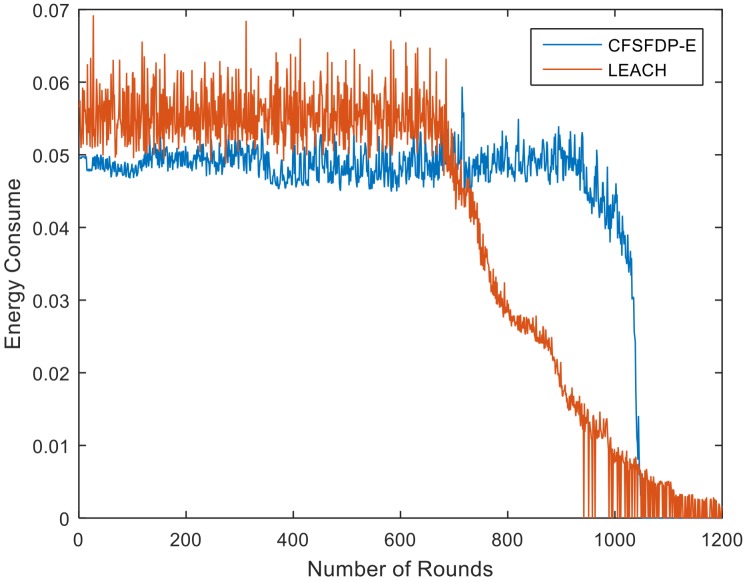
Energy consumption of LEACH and CFSFDP-E.

The proposed CFSFDP-E algorithm takes energy into account. When selecting CHs, we choose the nodes with high local density, large distance and high residual energy. The result shows that CFSFDP-E clustering algorithm can effectively postpone the FND. The FND of CFSFDP-E is 822, which is about nine times the 91 of CFDFDP. It is also longer than that of LEACH which means CFSFDP-E can balance the energy consumption of the networks effectively. Figure 3 shows the simulation results of these three algorithms. Figure 4 is the energy consumption of every round in LEACH and CFSFDP-E. Obviously, CFSFDP-E can keep most of the nodes alive in the network, which means it can balance the energy consumption more effectively than LEACH, so that the existence of the death of first node happens later.

### 5.3. Different Forms of Energy in CFSFDP-E

CFSFDP-E considers the residual energy, while the energy can have many forms that make the nodes with high residual energy more likely to become CHs than those with lower residual energy. In order to get a better performance of balancing energy consumption, we make some improvements on CFSFDP-E. Set $E_{f}$ to describe different forms of energy. The trends of curves $E_{f}={\left(1/\log E\right)}^{x}$ or $E_{f}=E^{y}$ can adjust the proportion of energy appropriately. Because when the expression of γ is changed from Equation (11) to $\gamma_{i}(r)=\rho_{i}(r){\left[1/\log E_{i}(r)\right]}^{x}\delta_{i}(r)$ or $\gamma_{i}(r)=\rho_{i}(r){\left[E_{i}(r)\right]}^{y}\delta_{i}(r)$, the impact of residual energy will be increased so that the possibility of nodes with high residual energy to be selected as cluster heads will increase.

In following experiments, Table 6 shows the results of different values of x,y for different forms of energy.

From the result, we can observe that when the form of energy is $E^{y}$, the FND is 978 as y=3, which is more than 150 rounds longer than that of y=1. And when the form of energy is ${\left(1/\log E\right)}^{6}$, the FND, HND and LND is very close to each other which means the energy consumption of all nodes is in a state of equilibrium. In addition, it is obvious that the performance of FND is better as the order increases for both forms of energy. The reason for this result is that when the order of energy increases, the impact of energy will hold the dominate position, while the influence of local density and distance will be less and less. In order to balance the impact of local density, distance and energy, it is indispensable to normalize these three elements.

We set $\gamma^{\prime }$ as the γ after normalization. $\gamma^{\prime }$ in the r round for node i can be calculated as follows:

(15) $$\begin{array}{cc} \gamma_{i}^{\prime }(r) & =\rho_{i}^{\prime }(r)E_{i}^{\prime }(r)\delta_{i}^{\prime }(r)\\ & =\frac{\rho_{i}(r)-\rho_{\min}(r)}{\rho_{\max}(r)-\rho_{\min}(r)}\cdot \frac{E_{i}(r)-E_{\min}(r)}{E_{\max}(r)-E_{\min}(r)}\cdot \frac{\delta_{i}(r)-\delta_{\min}(r)}{\delta_{\max}(r)-\delta_{\min}(r)}\;i\in I \end{array}$$

where $\rho_{i}^{\prime }(r),E_{i}^{\prime }(r),\delta_{i}^{\prime }(r)$ represent the local density, residual energy and distance of each node after normalization in the *r* round, respectively:

(16) $$\left\{ \begin{array}{l} \rho_{\min}(r)=\min[\rho_{i}(r)],\;i\in I\\ \rho_{\max}(r)=\max[\rho_{i}(r)],\;i\in I\\ E_{\min}(r)=\min[E_{i}(r)],\;i\in I\\ E_{\max}(r)=\max[E_{i}(r)],\;i\in I\\ \delta_{\min}(r)=\min[\delta_{i}(r)],\;i\in I\\ \delta_{\max}(r)=\max[\delta_{i}(r)],\;i\in I \end{array}\right.$$

In the first round, the initial energy of all nodes is the same. Therefore, we do not normalize the energy and the value of $\gamma_{i}$ is still calculated by Equation (5) for each node in this round. Table 7 is the result of different forms of energy after normalization.

We can observe that after normalization, the results in Table 7 are close to each other, which means that different forms of energy don’t make much difference in the results.

Considering the simplicity and performance, we choose $E_{f}=E^{2}$ as the form of energy in the following experiments, then the value of $\gamma_{i}^{\prime }(r)$ for node i in the round r after normalization should be calculated by $\gamma_{i}^{\prime }(r)=\rho_{i}^{\prime }(r){E_{f,}}_{i}^{\prime }(r)\delta_{i}^{\prime }(r)$. ${E_{f,}}_{i}^{\prime }(r)$ is defined as:

(17) $${E_{f,}}_{i}^{\prime }(r)=\frac{E_{i}^{2}(r)-\underset{j\in I}{\min}[E_{j}^{2}(r)]}{\underset{j\in I}{\max}[E_{j}^{2}(r)]-\underset{j\in I}{\min}[E_{j}^{2}(r)]}$$

Thus, the values of density-energy after normalization can be calculated by ${\omega_{i}}^{\prime }(r)={\rho_{i}}^{\prime }(r){E_{f,}}_{i}^{\prime }(r)$, and they should be listed in descending order to determine the distance of every node and allocation of CMs.

### 5.4. Dynamic Threshold η

In the CFSFDP algorithm, the threshold η is fixed. However, in a WSN, the local density, distance and energy of each node are changing as the number of rounds increases. Therefore, it is necessary to choose an appropriate dynamic threshold to determine the number of cluster heads. According to LEACH, we set p = 0.1, which represents the possibility of being selected as cluster head is 0.1. In the experiments above, we choose 10% of the number of surviving nodes as the number of cluster heads. It is not an optimal threshold for this algorithm. It’s necessary to choose a dynamic threshold to deal with three changeable factors.

Firstly, we set $t_{dc}(r)=[d_{c}(r)-\delta_{\min}(r)]/[\delta_{\max}(r)-\delta_{\min}(r)]$, which represents the normalization of cutoff distance, where $\delta {\left(r\right)}_{\min},\;\delta {\left(r\right)}_{\max}$ is the minimum and maximum value of distance in the current round explained in (16). The dynamic threshold should also take residual energy, local density and distance into consideration. In CFSFDP-E algorithm, we have already set $\omega^{\prime }=\rho^{\prime }{E_{f}}^{\prime }$. Thus, we define dynamic threshold as Equation (18):

(18) $$\eta (r)=\alpha \cdot \omega_{\max}^{\prime }(r)\cdot t_{dc}(r)$$

where α is a constant to determine the threshold. $\omega_{\max}^{\prime }(r)$ represents the maximum value of $\omega^{\prime }(r)$ in the current round, that is, $\omega_{\max}^{\prime }(r)=\max[\omega^{\prime }(r)]$. In order to get a better performance of WSN, Table 8 shows the performance of network lifetime with different α.

**Figure 5:**
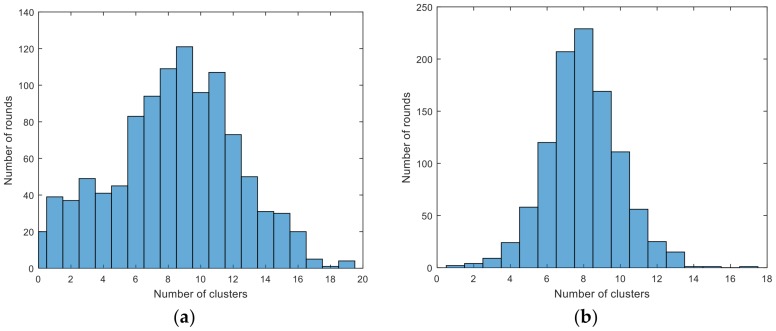
Distributions of the number of cluster heads in CFSFDP-E and LEACH. (**a**) distribution of the number cluster heads in CFSFDP-E. (**b**) distribution of the number of cluster heads in LEACH.

When the value of the threshold is too small, the number of nodes with the $\gamma_{i}$ above the threshold is large, resulting in the formation of too many clusters. In the contrast, if threshold is too large, there will be little or no clusters in the network. Both situations will cause more energy consumption. From Table 8, the performance of different values of α varies from each other. By comparing with the previous experiments, we choose α=2, which means the dynamic threshold of this experimental scenario is $\eta =2\omega_{\max}^{\prime }t_{dc}$. Figure 5 describes distributions of the number of cluster heads in CFSFDP-E and LEACH. In the LEACH protocol, the number of clusters is almost between 6 and 12, and in the CFSFDP-E algorithm, the most densely distributed region is 6 to 10, which is similar to LEACH. Therefore, the dynamic and adaptive threshold is suitable for WSNs.

### 5.5. Comparative Experiments

The proposed CFSFDP-E algorithm takes local density, distance and residual energy into consideration to get the appropriate number of clusters and choose suitable cluster heads. Based on the experiments above, we choose $E_{f}=E^{2}$ as the form of energy. The normalization of density-energy ω and γ can be calculated by $\omega_{i}^{\prime }(r)=\rho_{i}^{\prime }(r){E_{f,}}_{i}^{\prime }(r)$, $\gamma_{i}^{\prime }(r)=\rho_{i}^{\prime }(r){E_{f,}}_{i}^{\prime }(r)\delta_{i}^{\prime }(r)$ for each node in the r round, respectively. The dynamic threshold is set to $\eta (r)=2\omega_{\max}^{\prime }(r)t_{dc}(r)$.

In order to demonstrate the performance of CFSFDP-E algorithm, several protocols will be compared in this part. The results shown below are all the average values of 20 independent experiments.

**Figure 6:**
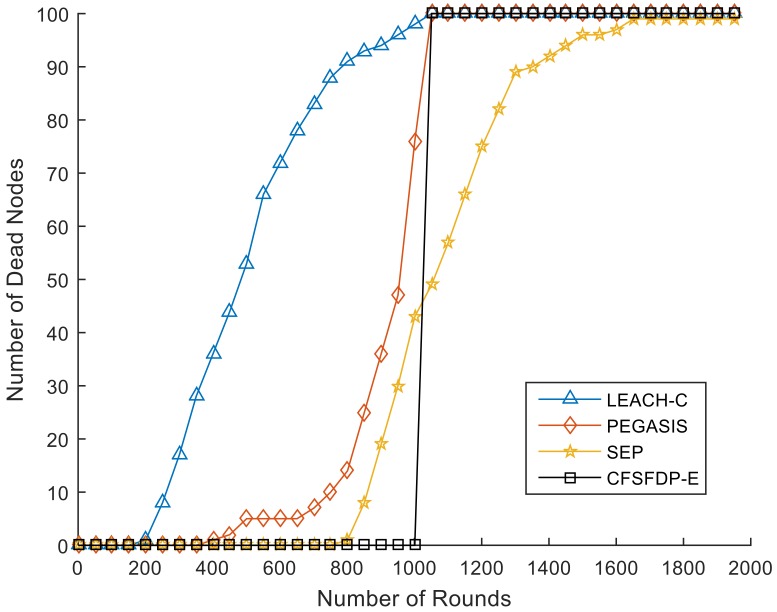
Network lifetime comparison of LEACH-C, PEGASIS, SEP and CFSFDP-E.

Figure 6 shows the network lifetime comparison of LEACH-C [10], PEGASIS [11], SEP [12] and CFSFDP-E. The first three protocols are famous and classical. In SEP protocol, there are a percentage of numbers of sensor nodes is equipped with additional energy resources. In this experiment, we set 10 nodes with double energy compared to normal nodes. The area size, number of nodes, location of sink node, length of data packets and control packets and other basic simulation parameters are all the same as those in Table 4.

From Figure 6, we can also observe that the FND of CFSFDP-E is larger than that of the other protocols, which means it can keep all the nodes alive in most of rounds and the death for first node is later. Experiments prove that the performance of CFSFDP-E algorithm is better than these classical protocols, indicating that the proposed approach can balance the energy consumption of the network.

**Figure 7:**
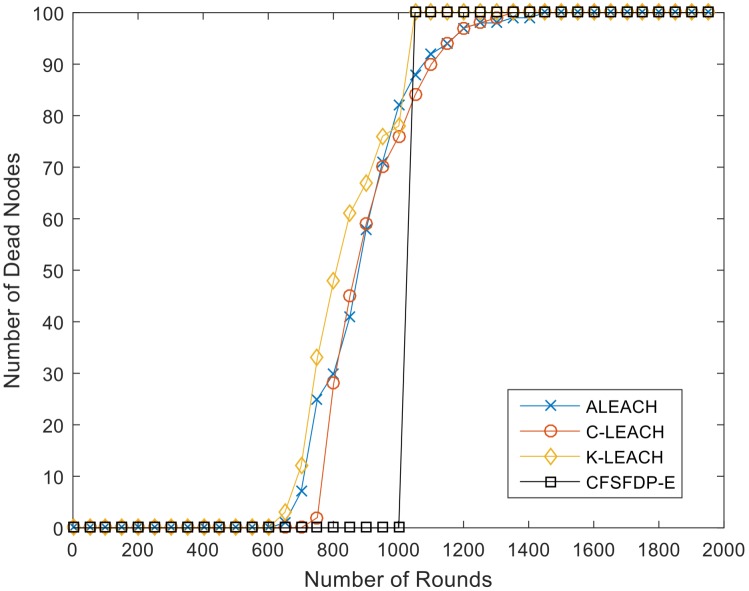
Network Lifetime comparison of ALEACH, C-LEACH, K-LEACH and CFSFDP-E.

The protocols of ALEACH [17], C-LEACH [18] and K-LEACH [19] are improvements based on LEACH. They optimize the clustering division and cluster heads selection. ALEACH, C-LEACH and K-LEACH select nodes with high residual energy as cluster heads with different forms of threshold T(i) shown in (14). ALEACH takes nodes’ residual energy and maximum energy into consideration while C-LEACH and K-LEACH considers residual energy and initial energy with different forms to choose clusters heads. Figure 7 is the result for these four protocols and CFSFDP-E algorithm with same parameters in the same experimental scenario shown in Table 4.

**Figure 8:**
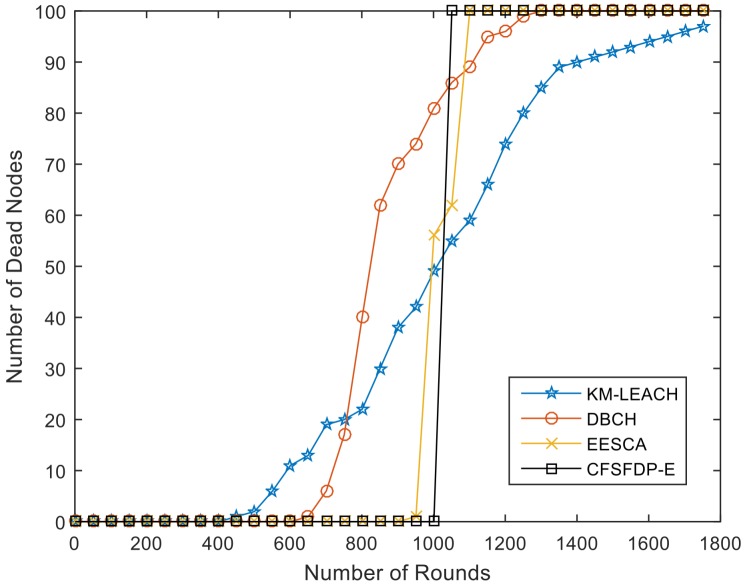
Network Lifetime Comparison of KM-LEACH, DBCH, EESCA, and CFSFDP-E.

It is obvious that the death of first node of the three improved protocols based on LEACH are all less than 800 rounds, while the FND of CFSFDP-E is over 1000. When first node dies, the residual energy of all nodes will be exhausted in hundreds of rounds. Table 9 and Figure 8 show a network lifetime comparison of the proposed CFSFDP-E, KM-LEACH [3], DBCH [20] and EESCA [21].

KM-LEACH (KMEANS-LEACH) uses the k-means clustering algorithm to form clusters and chooses cluster heads which are the nearest to the centers. The Distance-Based Cluster Head (DBCH) algorithm establishes a new threshold which includes the node energy and distance between node and sink node and distance between cluster head and sink node for measuring the threshold value.

The Energy Efficient Structured Clustering Algorithm (EESCA) is a hybrid cluster head selection method with parameter location centrality and nodes’ residual energy on the fixed clusters [21]. The area of deployment is exactly divided into four regions based on coordinates. Initially, the nodes which have the minimum average communication distance with other nodes are selected as cluster heads and remain their role until the cluster heads lose its 50% of their energy. This process continues until all the nodes in the cluster lose 50% of their total energy. And the remaining cluster head selection process is based on the residual energy of the nodes. Node which has the higher residual energy acts as the cluster head for the corresponding rounds.

These algorithms are simulated in the same experimental situations to compare the performance. From Figure 8, it is obvious that CFSFDP-E algorithm outperforms KM-LEACH, DBCH and EESCA in terms of energy consumption equilibrium. The FND of CFSFDP-E is 1003, which is over 2 times higher than KM-LEACH, 1.6 times than DBCH and over 50 rounds larger than EESCA. We can find that the result of EESCA is very close to our proposed algorithm CFSFDP-E. However, the clustering scheme of EESCA is inflexible. It always divides all the nodes into four clusters based on their coordinates, while the CFSFDP-E algorithm divides various clusters based on changing local density, residual energy and distance of each living node in every round.

**Figure 9:**
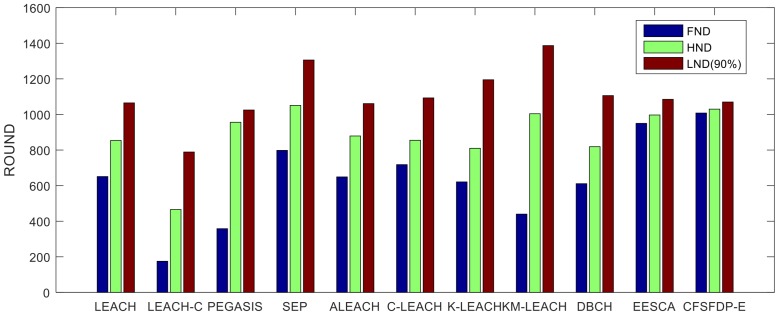
Network lifetime comparison of LEACH, LEACH-C, SEP, ALEACH, C-LEACH, K-LEACH, KM-LEACH, DBCH, EESCA, and CFSFDP-E.

From the analysis above, our proposed CFSFDP-E algorithm can balance energy consumption effectively based on every node’s local density, distance and residual energy. Figure 9 shows FND, HND and LND for above algorithms. It is proved that our proposed CFSFDP-E algorithm prolongs the life span of the first dead node and half dead nodes compared to these related algorithms.

## 6. Conclusions

In this paper, we propose an energy balanced CFSFDP-E algorithm protocol based on CFSFDP. It considers local density, distance and residual energy when selecting cluster heads in WSN. The nodes with high local density and high residual energy will be chosen as cluster heads. To get better performance, we also discuss about different forms of energy to balance energy consumption effectively and we determine $E^{2}$ as the form of energy. In addition, in order to balance the influence of three factors, we use parameters after normalization in the simulation experiments. Due to the changing parameters of every round, we set a dynamic threshold to determine an appropriate number of cluster heads. Experiments have demonstrated that CFSFDP-E algorithm can delay the number of rounds before the first node’s death. The round when the first node dies is prolonged by 50% compared to LEACH and it also outperforms several classical protocols (LEACH-C, PEGASIS and SEP), improved LEACH and KM-LEACH protocols (ALEACH, C-LEACH and K-LEACH) and protocols proposed in recent years (DBCH and EESCA. All these results show that the CFSFDP-E algorithm has superiority in terms of energy-balance, the total energy consumption and the number of living nodes.

The main limitation of our proposed CFSFDP-E algorithm is that it cannot decide the number of cluster heads, which relies on the threshold we set for every round. In future works, we may further study the dynamic threshold for different simulation scenarios and multi-hop communication between cluster heads based on the CFSFDP-E algorithm to prolong the lifetime of the network.

## Figures and Tables

**Table 1:** The local density and distance of six nodes.

Nodes	a	b	c	d	e	f
ρ**(DESC)**	$\rho_{a}$	$\rho_{b}$	$\rho_{c}$	$\rho_{d}$	$\rho_{e}$	$\rho_{f}$
δ	$d_{af}$	$d_{ba}$	$d_{ca}$	$d_{da}$	$d_{ec}$	$d_{fe}$
**Class**	**1**	**1**	**2**	**1**	**2**	**2**

**Table 2:** The density-energy and distance (determined by local density) of six nodes.

Nodes	c	f	b	d	e	a
ω **(DESC)**	$\omega_{c}$	$\omega_{f}$	$\omega_{b}$	$\omega_{d}$	$\omega_{e}$	$\omega_{a}$
δ	$d_{ca}$	$d_{fe}$	$d_{ca}$	$d_{da}$	$d_{ec}$	$d_{af}$
**Class**	**1**	**?**	**2**	**\**	**\**	**?**

**Table 3:** The density-energy and distance (determined by density-energy) of six nodes.

Nodes	c	f	b	d	e	a
ω **(DESC)**	$\omega_{c}$	$\omega_{f}$	$\omega_{b}$	$\omega_{d}$	$\omega_{e}$	$\omega_{a}$
δ	$d_{cb}$	$d_{fc}$	$d_{bc}$	$d_{db}$	$d_{ef}$	$d_{ad}$
**Class**	**1**	**1**	**2**	**2**	**1**	**2**

**Table 4:** Experimental Parameters.

Parameter	Value	Parameter	Value
Area	100 m × 100 m	$E_{TX}$	50 nJ/bit
Sink Node	(50,150)	$E_{RX}$	50 nJ/bit
Initial Energy	0.5J	$\varepsilon_{fs}$	10 pJ/bit
Packet Length	4000 bits	$\varepsilon_{mp}$	0.0013 pJ/bit
Control Length	100 bits	$E_{DA}$	5 nJ/bit
p	0.1	$r_{\max}$	3000

**Table 5:** Network lifetime comparison of LEACH, CFSFDP and CFSFDP-E.

ALGORITHM	FND	HND	LND (90%)
LEACH	688	874	1075
CFSFDP	91	972	1765
CFSFDP-E	822	1009	1036

**Table 6:** Network lifetime with different forms of energy.

FORM OF ENERFGY	\	FND	HND	LND (90%)
$E_{f}=E^{y}$	*y* = 1	822	1009	1036
	*y* = 2	980	1000	1006
	*y* = 3	978	1001	1005
$E_{f}={\left(1/\log E\right)}^{x}$	*x* = 2	873	1006	1068
	*x* = 4	965	1005	1023
	*x* = 6	991	1014	1021

**Table 7:** Network lifetime with different forms of energy after normalization.

FORM OF ENERFGY	\	FND	HND	LND (90%)
$E_{f}=E^{y}$	*y* = 1	991	1017	1032
	*y* = 2	1005	1026	1030
	*y* = 3	1002	1021	1027
$E_{f}={\left(1/\log E\right)}^{x}$	*x* = 2	994	1018	1047
	*x* = 4	995	1019	1036
	*x* = 6	998	1020	1027

**Table 8:** Network lifetime with different α.

Threshold η	FND	HND	LND (90%)
10% alive nodes	1001	1019	1030
α=0.5	978	1002	1011
α=1	995	1016	1023
α=1.5	996	1017	1024
α=2	1003	1025	1031
α=2.25	1002	1027	1034
α=2.5	1000	1026	1033
α=2.75	999	1026	1033
α=3	993	1023	1030

**Table 9:** Network lifetime Comparison of KM-LEACH, DBCH, EESCA and CFSFDP-E.

ALGORITHM	FND	HND	LND (90%)
KM-LEACH	440	1004	1087
DBCH	611	819	1106
EESCA	948	997	1085
CFSFDP-E	1003	1025	1031
